# Study on Separation of Desulfurization Wastewater in Ship Exhaust Gas Cleaning System with Rotating Dynamic Filtration

**DOI:** 10.3390/membranes15070214

**Published:** 2025-07-18

**Authors:** Shiyong Wang, Juan Wu, Yanlin Wu, Wenbo Dong

**Affiliations:** 1 Department of Environmental Science and Engineering, Fudan University, Shanghai 200433, China; wbdong@fudan.edu.cn; 2 Shanghai Research Institute of Chemical Industry Co., Ltd., Shanghai 200062, China; wj@ghs.cn; 3 National Petroleum and Chemical Industry Filtration & Separation Engineering Research Center, Shanghai 200062, China; 4 School of Resources and Environmental Engineering, Shanghai Polytechnic University, Shanghai 201209, China; wuyanlin@fudan.edu.cn

**Keywords:** EGC desulfurization wastewater, rotating dynamic filtration, flux, turbidity, shear force, back pressure, energy consumption, pilot-scale validation

## Abstract

Current treatment methods for desulfurization wastewater in the ship exhaust gas cleaning (EGC) system face several problems, including process complexity, unstable performance, large spatial requirements, and high energy consumption. This study investigates rotating dynamic filtration (RDF) as an efficient treatment approach through experimental testing, theoretical analysis, and pilot-scale validation. Flux increases with temperature and pressure but decreases with feed concentration, remaining unaffected by circulation flow. For a small membrane (152 mm), flux consistently increases with rotational speed across all pressures. For a large membrane (374 mm), flux increases with rotational speed at 300 kPa but firstly increases and then decreases at 100 kPa. Filtrate turbidity in all experiments complies with regulatory standards. Due to the unique hydrodynamic characteristics of RDF, back pressure reduces the effective transmembrane pressure, whereas shear force mitigates concentration polarization and cake layer formation. Separation performance is governed by the balance between these two forces. The specific energy consumption of RDF is only 10–30% that of cross-flow filtration (CFF). Under optimized pilot-scale conditions, the wastewater was concentrated 30-fold, with filtrate turbidity consistently below 2 NTU, outperforming CFF. Moreover, continuous operation proves more suitable for marine environments.

## 1. Introduction

Ships are one of the most important modes of transportation due to their large carrying capacity and low operating costs, accounting for more than 90% of global trade by volume [1,2]. However, the combustion of petroleum fuels during navigation emits substantial quantities of air pollutants, including nitrogen oxides (NO_X_), sulfur oxides (SO_X_), particulate matter (PM), and greenhouse gases (GHGs) [3,4,5,6]. International organizations and national authorities have established regulations to prevent ship exhaust pollution [7,8]. For example, the International Maritime Organization (IMO) requires that marine fuel sulfur content not exceed 0.5% in global waters and 0.1% in four major emission control areas, effective from 1 January 2020 [9]. The installation of an exhaust gas cleaning (EGC) system is currently the most effective and economical solution to comply with these sulfur restrictions [10,11,12]. However, the wastewater generated during the EGC process must be treated in accordance with the Guidelines for an Exhaust Gas Cleaning System (Resolution MEPC.259 (68)) [13].

Unlike other ship wastewaters such as domestic sewage [14,15,16], oily wastewater [17,18,19], and ballast water [20,21,22], EGC desulfurization wastewater presents a new challenge. The pollutants are extremely fine, adhesive, and highly adsorptive, which necessitates high-precision filtration and results in high filtration resistance and severe membrane fouling. The minimum particle size is less than 2.0 μm, and the maximum resistance of the filter cake can reach 10^12^ m^−1^. Additionally, the treatment system must have a small footprint, be easy to operate, minimize safety risks, and reduce secondary waste in the marine environment. Currently, two main technologies are applied: centrifugation coupled with flocculation and tubular cross-flow filtration (CFF). The centrifugation–flocculation approach is sensitive to wastewater concentration, particle size, and flocculation performance, often leading to fluctuating removal efficiencies for fine particles, as well as issues such as a high flocculant dosage and safety risks due to rotational machinery. CFF can effectively remove fine pollutants but faces problems, including a high volume of residual concentrate, large space requirements, and high energy consumption.

Research into the treatment of ship exhaust desulfurization wastewater has developed relatively late and largely draws on technologies established for land-based desulfurization, such as coagulation, flotation, adsorption [23,24,25], hydrocyclone separation [26], CFF [27], or combinations of these methods [28]. Some studies have demonstrated the feasibility of meeting treatment targets, but these approaches are generally unsuitable for marine applications due to their process complexity, large footprint, complicated operation, and high operating costs.

Rotating dynamic filtration (RDF) is an emerging separation technology that offers a promising performance for fouling control, flux enhancement, and energy saving via rotation of the membrane element or other components. RDF can achieve shear rates of approximately 3 × 10^5^ s^−1^ [29,30], which effectively mitigates concentration polarization and reduces cake layer formation on the membrane surface. In microfiltration (MF) and ultrafiltration (UF) applications, the circulation flow in RDF represents only 3–5% of the feed, or 10–15% for nanofiltration (NF) and reverse osmosis (RO), whereas CFF requires a circulating flow 50–100 times the feed. RDF thus enables energy savings of approximately 60–80% compared to CFF [31,32,33]. Furthermore, RDF can efficiently concentrate high-strength feed streams to minimal residual volumes. For example, M. Chai et al. [34] reported that RDF achieved a permeate flux approximately 15 times higher than CFF and a 1.7-fold increase in concentration during yeast suspension separation. Current RDF research focuses on applications in fermentation [35], biology [36,37,38,39], beverages [40,41,42,43], food processing [44,45,46,47,48,49], and the treatment or resource recovery of special waste liquids [50,51,52,53], but studies on RDF for wastewater treatment remain limited. The underlying fluid dynamics including velocity distributions, back pressure, and shear force also require further clarification.

This study aims to apply rotating dynamic filtration (RDF) to the separation of EGC desulfurization wastewater from ships, with the objective of developing a suitable treatment method and elucidating the unique separation mechanisms. The effects of key variables including feed circulation flow, rotational velocity, membrane size, feed concentration, operating pressure, and temperature on flux and filtrate turbidity will be systematically investigated. To deepen the understanding of RDF separation mechanisms, the hydrodynamic principles governing the shear force and back pressure will be analyzed in detail. Finally, the long-term performance of RDF under optimized conditions will be validated through industrial-scale pilot testing.

## 2. Materials and Methods

### 2.1. Materials

**Figure 1:**
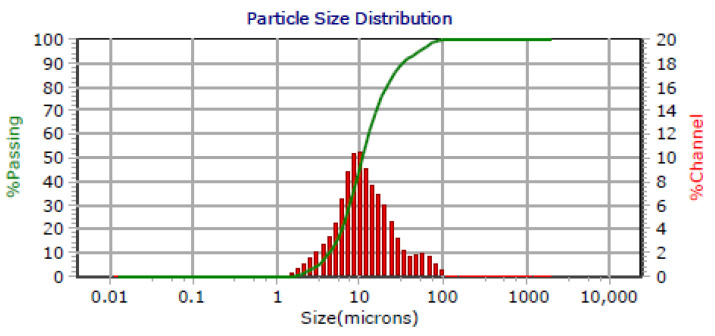
Particle size distribution of the experimental wastewater.

The raw feed wastewater used in this study was generated during ship bench engine EGC for desulfurization. Higher-concentration wastewater was produced through membrane concentration of the raw feed. The initial wastewater contained suspended solids at 266 mg·L^−1^, a density of 0.98 g·cm^−3^ at 20 °C, a turbidity of 499 NTU, a pH of 8.04, a COD of 855 mg·L^−1^, a PAH content of 198 μg·L^−1^, and a conductivity of 9.67 mS·cm^−1^. The particle size distribution, shown in Figure 1, ranged from approximately 1 to 100 μm, with a d_50_ value of 10.64 μm and a minimum particle size of 1.64 μm.

### 2.2. Experimental Device and Procedure

**Figure 2:**
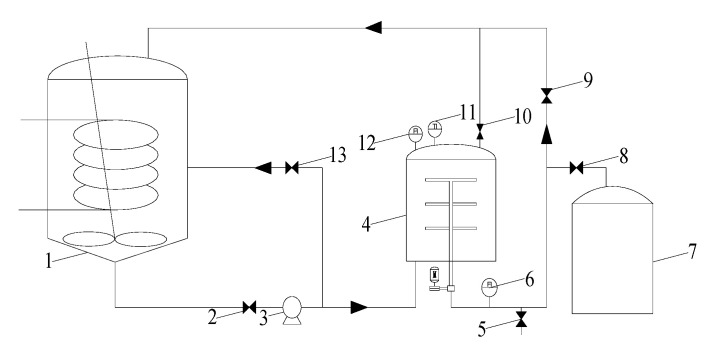
Process of the RDF experimental device. 1. Feed tank; 2. feed valve; 3. pump; 4. RDF filter; 5. sampling port; 6. flow meter; 7. permeate tank; 8. permeate valve; 9. permeate backflow valve; 10. feed backflow valve; 11. temperature sensor; 12. pressure sensor; 13. bypass control valve.

The experimental setup is shown in Figure 2 and consisted of a feed tank (equipped with stirring and heat exchange), pump (Zhejiang Alipu Technology Co., Ltd., Jinhua, China), RDF filter, permeate tank, temperature and pressure sensors (Shanghai Hengrui measurement & control Technology Co., Ltd., Shanghai, China), flow meter (Wuxi Jingfan Automation Instrument Co., Ltd., Wuxi, China), valves, pipeline, and control system. The core of the system was the RDF filter, comprising a hollow shaft, membrane element, and pressure vessel. Ceramic membrane disc elements from NOVOFLOW (Rain, Germany) were mounted on the hollow shaft inside the container. Key parameters for each membrane element are listed in Table 1. The membrane rotation was driven by a variable-frequency motor connected externally to the shaft. The rotational velocity ranged from 100 to 900 rpm for laboratory tests (one 0.035 m^2^ membrane element, 2 L container volume) and from 100 to 600 rpm for pilot tests (five 0.2 m^2^ membrane elements, 15 L container volume).

During operation, wastewater from the feed tank was pumped into the RDF filter. The concentrate was recirculated to the feed tank, while permeate was monitored by the flow meter and collected from the sampling port. To examine the influence of operational parameters, the pressure, rotational velocity, temperature, and circulation flow were controlled by adjusting the pump, motor frequency, heat exchanger, and valves.

### 2.3. Theory and Methods

#### 2.3.1. Determination of the Related Resistance

Darcy’s law applies to RDF, and the total resistance can be calculated as follows [54,55]:

(1) $$J=\frac{TMP}{\mu R_{t}}=\frac{TMP}{\mu (R_{m}+R_{c}+R_{f})}$$

where *TMP* is the transmembrane pressure, kPa; *µ* is the dynamic viscosity, mPa·s; *R_t_* is the total resistance, m^−1^; *R_m_* is the intrinsic membrane resistance, m^−1^; *R_c_* is the resistance from concentration polarization and the cake layer, m^−1^; and *R_f_* is the fouling resistance due to adsorption and blockage, m^−1^.

*R_m_* is calculated using clean water flux under differential pressure. *R_m_* + *R_f_* is determined after removing concentration polarization and deposited cake. *R_m_* + *R_f_* + *R_c_* is derived using feed flux, transmembrane pressure, and permeate viscosity.

#### 2.3.2. Determination of Back Pressure and Effective Pressure

Back pressure is a key hydrodynamic feature in RDF. When the membrane element rotates at high speed, centrifugal force generates reverse pressure on the permeate, weakening the effective transmembrane pressure. The magnitude of back pressure depends on the device structure, membrane element geometry and size, and rotational velocity.

The operating pressure (Δ*P*) in the RDF system is the sum of back pressure (*P_c_*) and transmembrane pressure difference (*TMP*):

(2) $$\Delta P=P_{c}+TMP$$

where Δ*P* is operating pressure, kPa; *P_c_* is back pressure, kPa; and *TMP* is the transmembrane pressure difference, kPa.

Back pressure weakens the effective transmembrane pressure, reducing permeate flux. It can be determined from the relationship between flux and rotational velocity at a constant operating pressure:

(3) $$P_{c}=\Delta P-TMP=\Delta P-\mu JR_{m}$$

where *J* is the flux, m^3^·m^−2^·s^−1^; *µ* is the liquid dynamic viscosity, mPa·s; and *R_m_* is the intrinsic membrane resistance, m^−1^.

Alternatively, back pressure can be calculated directly using the Bernoulli equation [56]:

(4) $$P_{c}=k^{2}\omega^{2}\frac{\rho }{4}(r_{i}^{2}+r_{o}^{2})$$

where *ρ* is the liquid density, kg·m^−3^; *r_i_* is the inner radius of the membrane element, m; *r_o_* is the outer radius of the membrane element, m; and *k* is the velocity factor, which reflects the degree of deviation from the ideal situation. The velocity factor *k* is influenced by the feed characteristics, membrane design, and membrane chamber structure and is typically determined experimentally. Because back pressure is inherent to RDF, the applied operating pressure must exceed the back pressure threshold to prevent reverse permeate flow.

#### 2.3.3. Evaluation of the Flow State

The Reynolds number (Re) is a dimensionless parameter that reflects the ratio of inertial to viscous forces in fluid flow. In the RDF process, Re is used to characterize the flow regime and is defined as follows:

(5) Re=kρωr2μ

where *k* is the velocity factor; *ρ* is liquid density, kg·m^−3^; *ω* is the rotating angular velocity of the membrane element, rad·s^−1^; *r* is the radius of the membrane element, m; and *μ* is the liquid dynamic viscosity, mPa·s. When the Re is less than 2.50 × 10^5^, the flow state is laminar, while when the *Re* is greater than 2.50 × 10^5^, the flow state is turbulent [57].

#### 2.3.4. Determination of Membrane Surface Shear Force

In the RDF system, rotation of the membrane elements induces a shear force on the membrane surface. The shear force under laminar and turbulent conditions can be estimated using the following expressions [58]:

(6) $$\tau_{l}=1.81\rho {\left(k\omega \right)}^{3/2}r\nu^{1/2}$$

(7) $$\tau_{t}=0.057\rho {\left(k\omega \right)}^{9/5}r^{8/5}\nu^{1/5}$$

where *τ_l_* is the shear force in laminar flow, Pa; *τ_t_* is the shear force in turbulent flow, Pa; *k* is the velocity factor; *ρ* is the liquid density, kg·m^−3^; *ω* is the rotating angular velocity of the membrane element, rad·s^−1^; *r* is the radius of the membrane element, m; and *ν* is kinematic viscosity, m^2^·s^−1^.

#### 2.3.5. Determination of Energy Consumption

In RDF, energy is mainly consumed by the motor driving the membrane elements, while in conventional cross-flow filtration (CFF), it is consumed by the feed pump for fluid circulation.

The energy consumption in RDF results primarily from overcoming the shear friction between the rotating membrane surface and the fluid and can be calculated as in [59].

(8) $$H_{R}=2\pi n\int_{0}^{R}\left(\tau_{u}+\tau_{d}\right)r^{2}\omega dr$$

where *n* is the number of membrane elements; *τ_u_* and *τ_d_* are the shear forces on two surfaces of membrane element, Pa; *τ_u_* and *τ_d_* are same in this experiment; *r* is the radius of the membrane element, m; and *ω* is the rotational velocity of the membrane element, rad·s^−1^.

The energy consumption of CFF is determined using the Bernoulli equation:

(9) $$H_{B}=P_{i}Q_{i}+P_{f}Q_{0}$$

where *P_i_* represents the pressure of the feed pump, kPa; *P_f_* is the operating pressure, kPa; *Q_i_* is the feed flow, m^3^·h^−1^; *Q*_0_ is the circulation flow, m^3^·h^−1^; *P_i_* is close to *P_f_*; and *Q_i_* is much smaller than *Q*_0_, so *P_i_Q_i_* can be ignored.

The specific energy consumption *U_R_*, defined as the energy consumed per unit volume of feed, is given by

(10) $$U_{R}=\frac{H_{T}}{V}$$

where *U_R_* is the specific energy consumption, W·m^−3^; *H_T_* represents the total energy consumption; and *V* represents the volume of feed material, m^3^.

## 3. Results and Discussion

### 3.1. Experimental Study on the Separating Behavior

The treatment capacity and purification performance are critical factors in the RDF process, as they are directly associated with permeate flux and filtrate turbidity. The process is influenced by multiple factors, including operating conditions, feed characteristics, and membrane properties.

#### 3.1.1. Influence of Feed Circulation

Membrane filtration typically enhances separation by promoting relative motion between the fluid and the membrane. In CFF, this motion is driven by high feed circulation, whereas in RDF, it is primarily generated by rapid membrane rotation.

**Figure 3:**
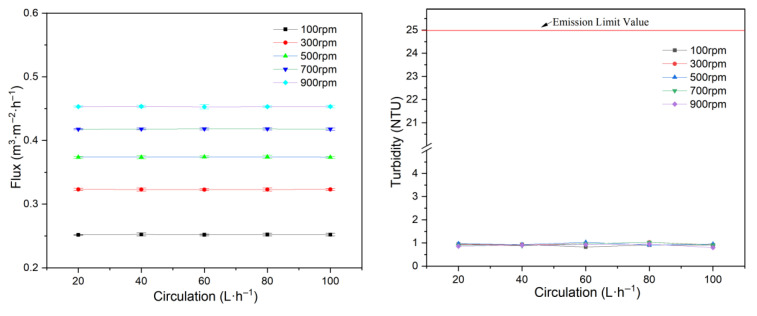
Effect of feed circulation on flux and filtrate turbidity.

Experiments examining flux and turbidity at various feed circulation rates were conducted using the 1# membrane disc, with a feed concentration of 266 mg·L^−1^, 200 kPa operating pressure, 20 °C, and rotational velocities ranging from 100 to 900 rpm. The results are presented in Figure 3.

Compared with the high-velocity motion resulting from membrane rotation, the effect of feed circulation is negligible. Thus, feed circulation has little influence on both flux and turbidity.

According to MEPC.259 (68), the turbidity of purified wastewater should not exceed 25 NTU. In all experiments, the filtrate turbidity remained below 1.5 NTU, indicating that the membrane element effectively removed almost all particulate contaminants and met regulatory requirements. Therefore, subsequent analysis does not focus on filtrate turbidity. For operational simplicity, the feed circulation was maintained at 60 L·h^−1^ in the following study.

#### 3.1.2. Influence of Membrane Element Rotational Velocity

Membrane rotation is a unique factor in RDF, directly affecting the separation process. Increasing membrane rotational velocity enhances the shear force at the membrane surface, thereby reducing filtration resistance and increasing flux. However, higher rotational speeds also generate back pressure, which can reduce the effective transmembrane pressure.

Flux and turbidity were investigated at rotational velocities from 100 to 900 rpm, using the 1# membrane disc, a feed concentration of 266 mg·L^−1^, pressures of 100–300 kPa, a feed circulation of 60 L·h^−1^, and 20 °C.

**Figure 4:**
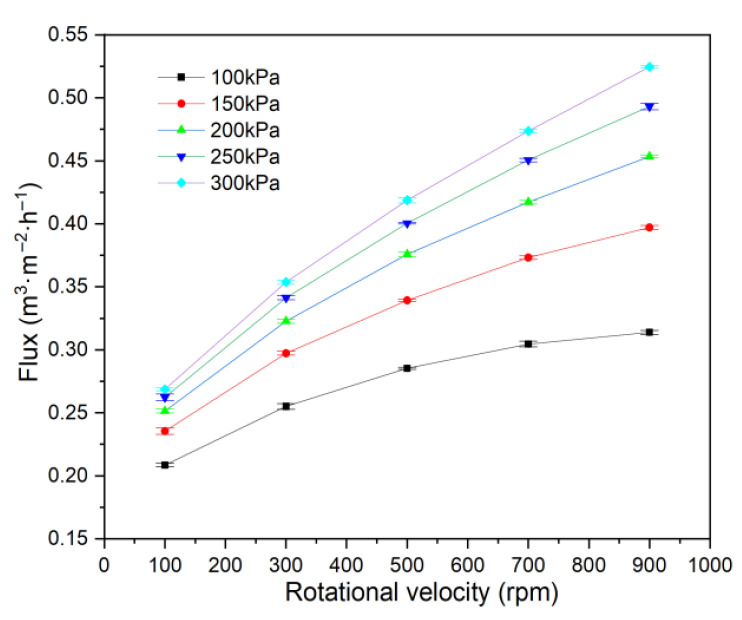
Effect of rotational velocity on flux.

As shown in Figure 4, flux increases with membrane rotational velocity at each pressure, but the rate of increase slows at higher velocities. The enhanced shear force generated by rotation partially removes particles deposited on the membrane surface, thereby mitigating filter cake formation and concentration polarization due to both active removal and reduced deposition. This effect reduces separation resistance and increases flux. However, at a high rotational velocity, additional particle removal becomes limited, so the rate of resistance reduction diminishes, resulting in a slower increase in flux.

The back pressure generated by membrane rotation is a characteristic phenomenon in RDF. As rotational velocity increases, so does back pressure. Although this back pressure reduces the effective driving force for filtration, the reduction in separation resistance caused by shear is more significant than the loss of driving pressure. Therefore, flux continues to increase with rotational velocity in these experiments.

#### 3.1.3. Influence of Membrane Element Size

Flow and pressure distributions in RDF vary with membrane disc radius, affecting permeate flux.

**Figure 5:**
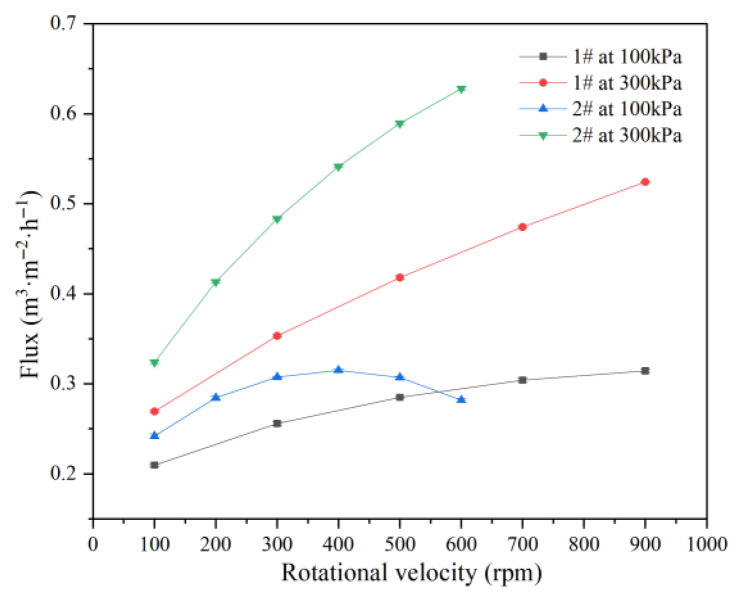
Effect of membrane size on flux.

Experiments were conducted using 1# and 2# membrane elements with identical precision but different diameters. The 1# membrane operated at 60 L·h^−1^ and the 2# membrane at 350 L·h^−1^, both with a feed concentration of 266 mg·L^−1^, pressures of 100 and 300 kPa, and at 20 °C. The results are shown in Figure 5.

Flux for the 1# and 2# membranes differed under an identical rotational velocity and pressure, mainly due to differences in shear force and back pressure associated with membrane size. Similar research work was carried out by B. John [60]. For the 1# membrane (152 mm diameter), flux increased with rotational velocity at both 100 and 300 kPa. For the 2# membrane (374 mm diameter), flux increased with rotational velocity at 300 kPa, but at 100 kPa, flux first rose and then declined over the 100–600 rpm range. At 300 kPa, the 2# membrane achieved higher flux than the 1# membrane at all velocities. At 100 kPa, the 2# membrane produced higher flux than the 1# membrane from 100 to 550 rpm, but lower flux above 550 rpm.

Rotation increases both shear force, which reduces resistance, and back pressure, which reduces the effective driving force. Larger membranes generate greater shear force, but also more back pressure. For the 1# membrane at both pressures and the 2# membrane at 300 kPa, the reduction in resistance outweighs the decrease in driving force, resulting in increased flux with higher rotational velocity. However, for the 2# membrane at 100 kPa, resistance reduction dominates from 100 to 550 rpm, but above 550 rpm, the loss in driving force becomes dominant, leading to a decrease in flux at higher velocities.

#### 3.1.4. Influence of Operating Pressure

**Figure 6:**
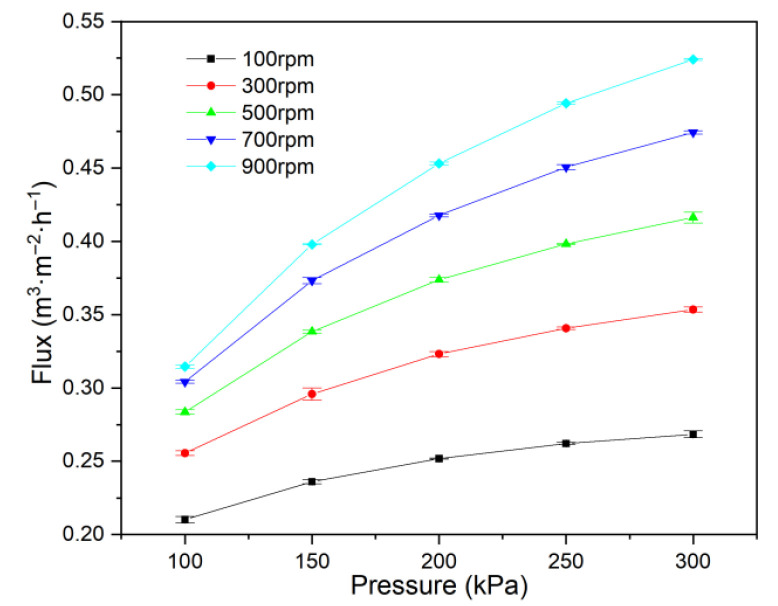
Effect of pressure on flux.

Operating pressure directly determines the driving force for RDF and significantly influences flux. The effect of operating pressure on flux was assessed across a range from 100 to 300 kPa, using the 1# membrane element under conditions of 266 mg·L^−1^ feed concentration, 60 L·h^−1^ circulation, 20 °C, and rotational velocities of 100–900 rpm. The experimental results are presented in Figure 6.

As shown in Figure 6, permeate flux increases steadily with operating pressure, but the rate of increase diminishes at higher pressures. Elevating the operating pressure increases the transmembrane pressure, promoting higher flux. However, increased pressure also leads to more particulate deposition on the membrane surface, which intensifies concentration polarization and cake layer formation. This additional resistance causes the rate of flux increase to slow at higher pressures. Because the manufacturer recommends a maximum operating pressure of 300 kPa, a suitable operating pressure for this study is 200–250 kPa.

#### 3.1.5. Influence of Feed Concentration

During EGC desulfurization wastewater separation, continuous discharge of purified liquid and concentration of the remaining wastewater require a clear understanding of how the feed concentration affects separation behavior in RDF.

The volume reduction rate (*VRR*), defined as the ratio of initial to concentrated volume, quantifies the concentration [61] and is calculated as follows:

(11) $$VRR=\frac{V_{f}}{V_{c}}$$

where *VRR* is the volume reduction rate; *V_f_* refers to the initial wastewater volume, m^3^; and *V_c_* is the volume of concentrated wastewater, m^3^.

**Figure 7:**
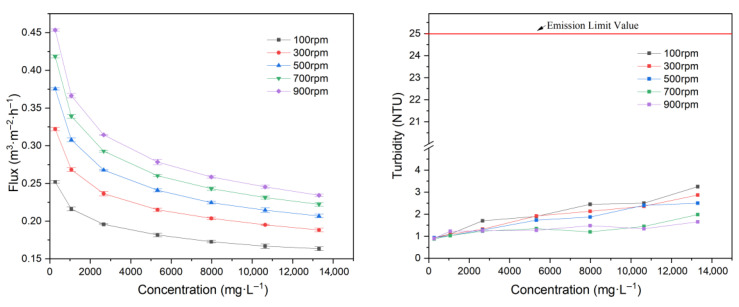
Effect of concentration on flux and filtrate turbidity.

In these experiments, the relationship between flux and feed concentrations of 266, 1064, 2660, 5320, 7980, 10,640, and 13,300 mg·L^−1^ was investigated, corresponding to concentration multiples (*VRR*) of 1, 4, 10, 20, 30, 40, and 50, respectively. As shown in Figure 7, permeate flux drops sharply at low concentrations, but the decline slows as the concentration exceeds approximately 5320 mg·L^−1^.

With an increasing feed concentration, more particulates are retained on the membrane, leading to the rapid formation of concentration polarization and cake layers, which significantly raises resistance and sharply reduces flux. However, due to high shear at the membrane surface, the thickness of these layers cannot increase indefinitely, resulting in a slower increase in resistance and a more gradual decrease in flux at high concentrations.

Figure 7 shows that permeate turbidity rises with feed concentration, but does not exceed 3.25 NTU, demonstrating that even at a high concentration, the membrane efficiently removes particulates. The increase in the turbidity with feed concentration was due to there being more particulates deposited on the membrane surface.

**Figure 8:**
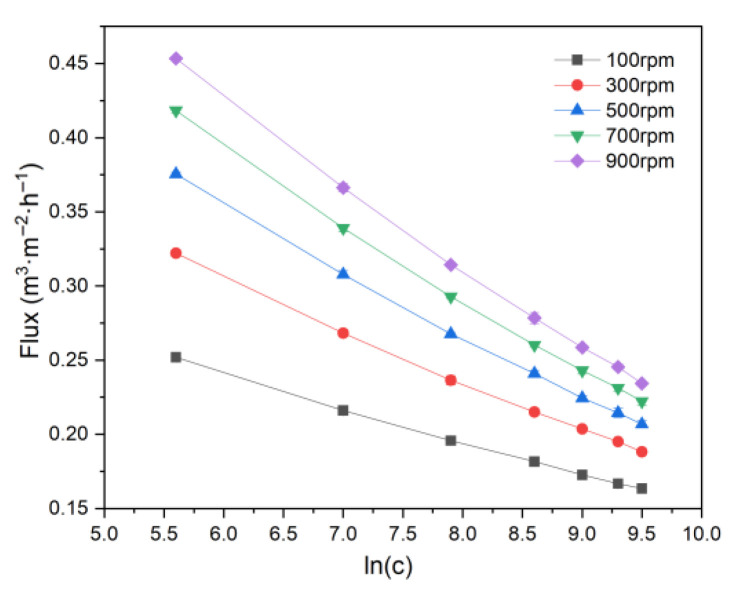
Logarithm of concentration vs. flux.

In addition, as shown in Figure 8, permeate flux decreases linearly with the logarithm of feed concentration, consistent with previous findings on RDF separation of soymilk [62].

*VRR* is a critical parameter in EGC wastewater treatment, as it defines the endpoint of filtration and the residual volume of concentrated wastewater. A higher *VRR* means less residual wastewater but leads to greater membrane fouling and lower flux. *VRR* should be selected based on practical needs. In these experiments, an optimal *VRR* of approximately 20 was identified, at which point the flux is reduced to a lower level and most of the liquid has been treated.

#### 3.1.6. Influence of Operating Temperature

Increasing temperature reduces fluid viscosity and enhances the diffusion of particles and solutes, both of which help alleviate concentration polarization, cake layer formation, and membrane fouling, thereby maintaining higher flux [63].

**Figure 9:**
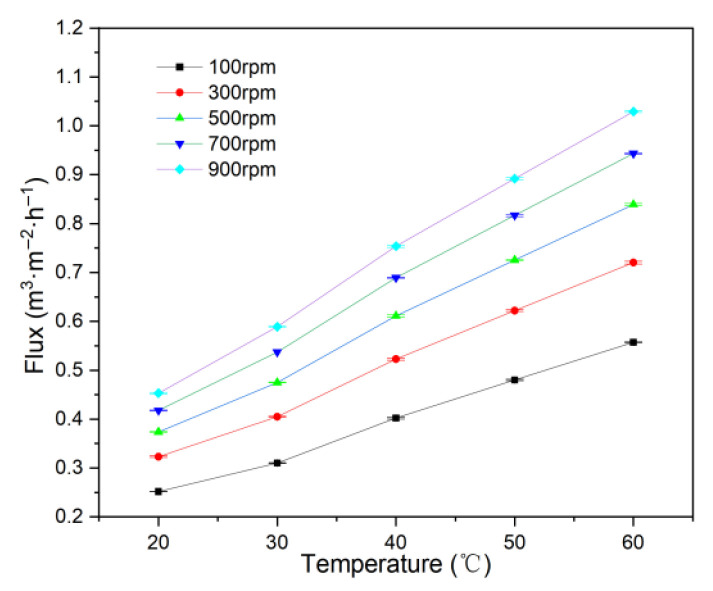
Effect of temperature on flux.

Experiments were conducted at temperatures from 20 °C to 60 °C, using the 1# membrane, 60 L·h^−1^ feed circulation, 266 mg·L^−1^ concentration, 200 kPa pressure, and rotational velocities of 100, 300, 500, 700, and 900 rpm. The results are shown in Figure 9.

Figure 9 demonstrates that increasing temperature improves permeate flux at all rotational velocities. According to Darcy’s law, permeate flux is proportional to the driving pressure and inversely proportional to fluid viscosity and total resistance. As temperature increases from 20 °C to 60 °C, wastewater viscosity decreases from 1.33 mPa·s to 0.64 mPa·s, which is likely the main reason for enhanced flux. Thus, raising temperature is recommended as an effective approach to increase wastewater treatment capacity without substantial additional energy consumption.

### 3.2. Theoretical Analysis of the Hydrodynamics in the RDF Process

#### 3.2.1. Determination of Average Back Pressure and Velocity Factor

When the membrane disc rotates at high velocity, the permeate inside the membrane rotates as well, resulting in back pressure induced by centrifugal force in the RDF process. The back pressure is mechanical counter-pressure from rotation, which will reduce the transmembrane pressure. The water flux experiments were designed in the different rotational velocities with 1#, 2#, 3#, and 4# membranes at 100 kPa, and then the back pressure and effective pressure at various rotational velocities can be calculated using Equation (3). The velocity factor *k* can be calculated by Equation (4), where back pressure, fluid density, membrane disc size, and rotational velocity are readily determined. Note that both back pressure and effective pressure exhibit gradients across different membrane positions, and the back pressure reported here represents the average value.

**Figure 10:**
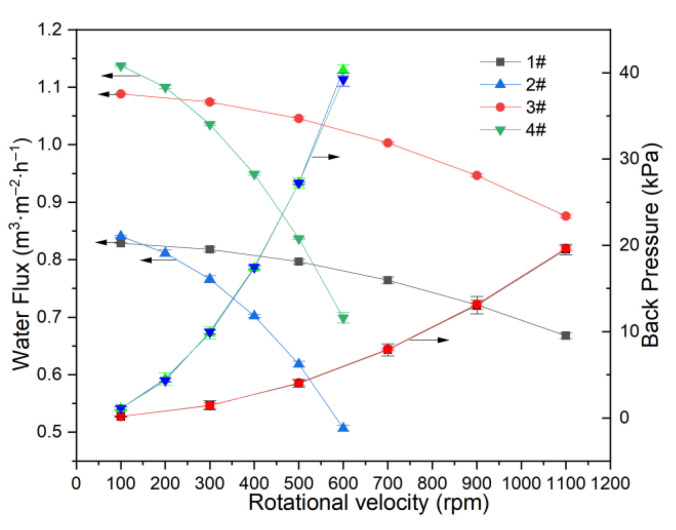
Water flux and back pressure in the RDF.

In this study, water fluxes for the four membrane elements were measured at 20 °C and 100 kPa across different rotational velocities. The resulting average back pressures, calculated by Equation (3), are shown in Figure 10.

Fluxes for all four membrane elements decrease as rotational velocity increases, with larger membrane elements exhibiting more pronounced declines. Average back pressures rise with rotational velocity and are more strongly affected in larger membranes. For membranes of the same size but different precision, average back pressure remains nearly identical. At a given rotational velocity, the larger membrane element exhibits greater average back pressure than the smaller one. The maximum average back pressure for the small membrane element is approximately 20 kPa at 1100 rpm, while for the larger element, it reaches about 40 kPa at 600 rpm.

The velocity factor *k* is determined from the experimentally obtained back pressure, inner and outer diameters of the membrane, rotational velocity, and fluid density. For the small membrane elements (1# and 3#), *k* equals 0.98, while for the larger elements (2# and 4#), *k* is 1.01. The value of *k* is influenced by the structure and dimensions of the separation chamber and the shape and size of the membrane. Previous studies report *k* values of 0.9 with eight-blade propellers on a fixed membrane [64], and 1.13 for rotating membranes [65]. The velocity factor exerts a substantial effect under conditions of high rotational speed or a large membrane size.

#### 3.2.2. Study on the Velocity of the Membrane and the Flow Status

**Figure 11:**
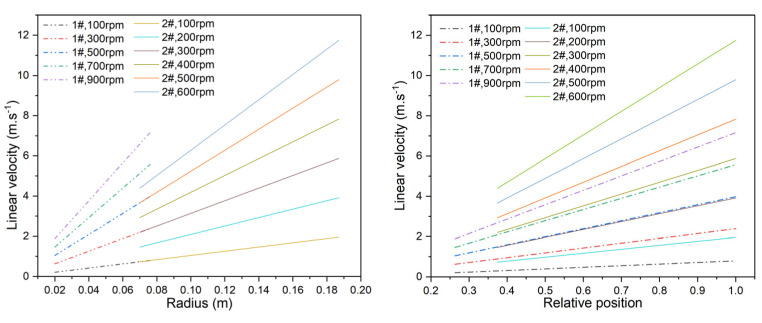
Linear velocity on the membrane surface at different rotational velocities.

Figure 11 presents the linear velocity distributions for the 1# and 2# membranes along the radius, both in absolute terms and relative to membrane position. Linear velocity increases with radius, reaching its maximum at the membrane edge. At 900 rpm, the edge linear velocity of the 1# membrane is 7.16 m·s^−1^, while at 600 rpm, the edge velocity of the 2# membrane is 11.74 m·s^−1^. The velocity distribution range of the larger membrane is narrower, and the velocity gradient across the surface is smaller.

**Figure 12:**
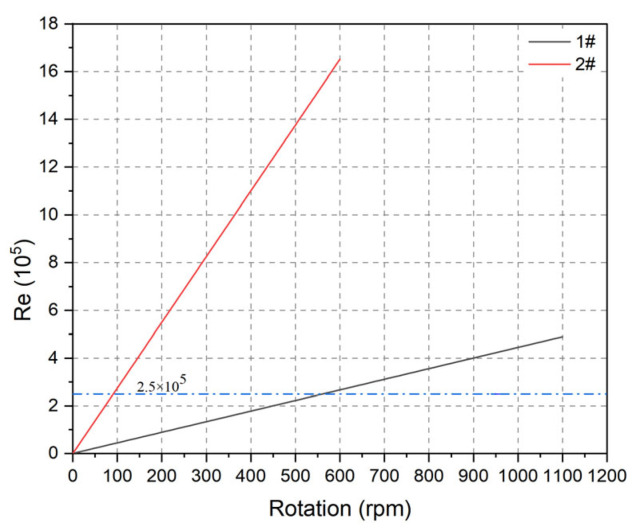
Reynolds numbers for the two membranes at different rotational velocities.

As shown in Figure 12, the Reynolds numbers for the 1# and 2# membranes at different rotational velocities were calculated using Equation (5). The Reynolds number is directly proportional to rotational velocity and to the square of the membrane radius; thus, the value for the 2# membrane is higher than for the 1# membrane at the same velocity. The 2# membrane reaches a Reynolds number of 2.75 × 10^5^, indicating turbulent flow, at 100 rpm, while the 1# membrane requires 560 rpm to reach this threshold.

It is generally preferable to operate under turbulent conditions for effective filtration. However, practical considerations, such as membrane durability and energy consumption, require that membrane size and rotational velocity be balanced. It is recommended to exceed this value of 2.75 × 10^5^ in this study.

#### 3.2.3. Distribution of Back Pressure and Effective Pressure Difference

Back pressure is a distinctive feature of the RDF process. As the rotational velocity of the membrane increases, the relative motion between the fluid and membrane surface enhances the removal of deposited particulates, but also increases back pressure, thereby reducing the effective pressure difference.

**Figure 13:**
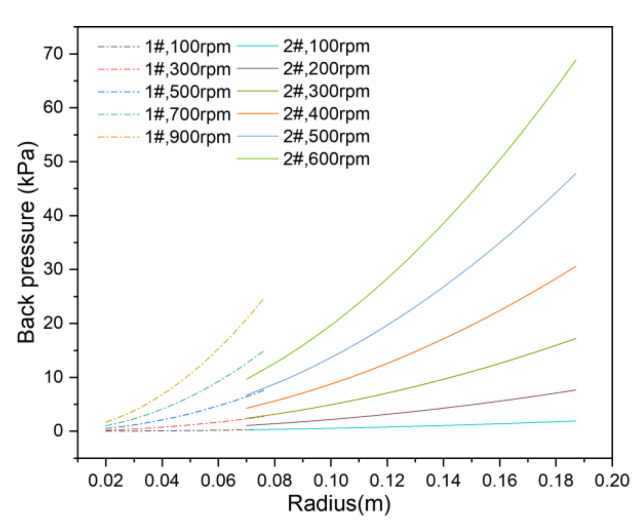
Back pressure on the membrane surface at different rotational velocities.

The average back pressure across the membrane was previously determined. Using Equation (4), the distribution of back pressure across the membrane surface at various velocities was calculated for the 1# and 2# membranes, as presented in Figure 13.

As rotational velocity increases, back pressure rises along the radial direction, with the highest value occurring at the membrane edge. For the 1# membrane at 900 rpm, the maximum back pressure at the edge is 24.56 kPa, with an average of 13.13 kPa. For the 2# membrane at 600 rpm, the maximum back pressure is 68.82 kPa, with an average of 39.23 kPa.

**Figure 14:**
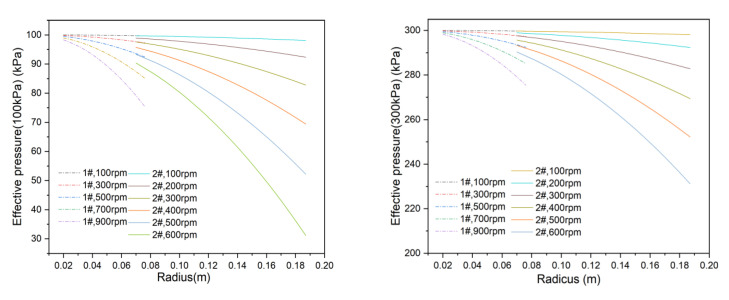
Effective pressure on the membrane surface at different rotational velocities.

In addition, the effective pressure difference for the 1# and 2# membranes under 100 kPa and 300 kPa operating pressures was also evaluated. As shown in Figure 14, the effective pressure difference decreases with increasing rotational velocity and radius. For the 2# membrane, the effective pressure difference is reduced to 31.18 kPa at 100 kPa and 600 rpm, which is less than one-third of the original operating pressure. The effective pressure difference in the 2# membrane shows more pronounced changes due to its larger size.

When comparing the two operating pressures, the effect of 100 kPa on the effective pressure difference is more significant than that of 300 kPa. Therefore, increasing the operating pressure is an effective approach to mitigate the influence of back pressure. Additionally, the operating pressure should always exceed the maximum back pressure to avoid permeate reverse flow.

#### 3.2.4. Shear Force on Membrane Surface and Its Effect on Filtration Process

Although rotation induces back pressure, it also generates shear force at the membrane surface, which is beneficial for separation performance. This is a key feature distinguishing the RDF process from other membrane processes.

Membrane rotation alters the hydrodynamic state, increases shear force, suppresses cake layer formation, enhances antifouling performance, and improves permeate flux [66]. Based on Equations (6) and (7), the shear forces on the 1# and 2# membranes were calculated at various membrane positions and rotational velocities. For a membrane disc, shear force increases progressively along the radius, reaching its minimum at the inner section and maximum at the edge.

**Figure 15:**
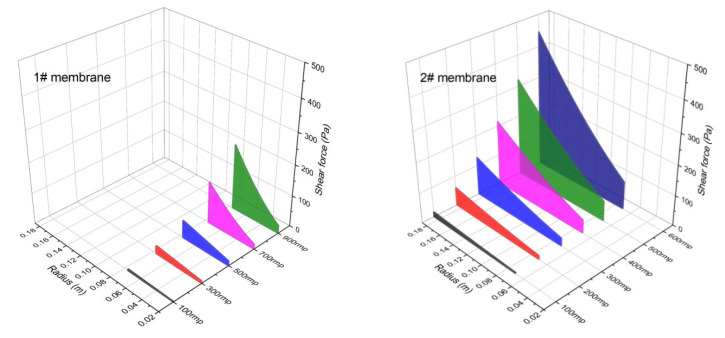
Shear forces at different positions of 1# and 2# membrane elements.

As shown in Figure 15, shear forces for both membranes increase with rotational velocity and radial position, and larger membranes generate greater shear force. The maximum shear force for the 1# membrane is 27.05 Pa, while the 2# membrane reaches 128.64 Pa at 300 rpm. Thus, using a larger membrane element is also an effective method to increase shear force at the membrane surface.

**Figure 16:**
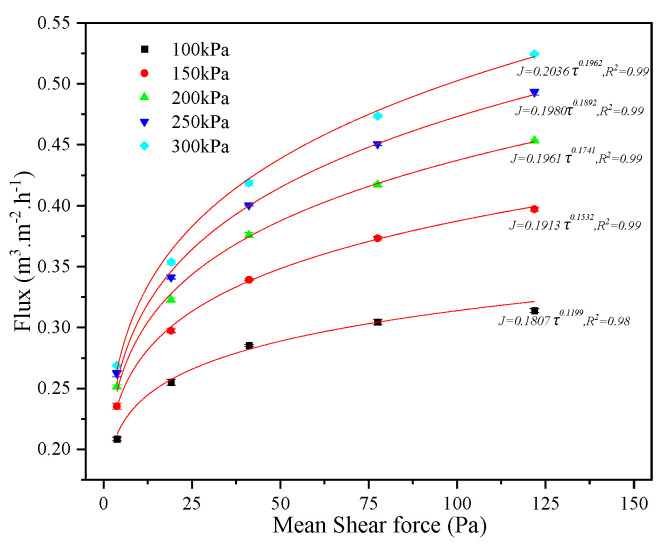
Fitting relationship between shear force and flux.

The relationship between average shear force and flux was investigated using EGC desulfurization wastewater with the 1# membrane, 266 mg·L^−1^, 20 °C, and various operating pressures and rotational velocities. As illustrated in Figure 16, flux increases with rising average shear force, although the rate of increase slows gradually. At the same average shear force, flux is higher under a higher operating pressure. Furthermore, the relationship between average shear force and flux follows a power function in this study, consistent with the findings of Z.H. Tu et al. [67] in the separation of calcium carbonate by a rotating membrane.

#### 3.2.5. Comparison of Energy Consumption Between RDF and CFF

The energy consumption of RDF and CFF was compared under the conditions of an equivalent flux, membrane area, and surface velocity.

Equivalent velocity and specific energy consumption were calculated using Equations (8) and (10) for the 1# membrane element (0.035 m^2^), 266 mg·L^−1^, 20 °C, 60 L·h^−1^ feed circulation, and rotational velocities of 300, 500, and 700 rpm.

The corresponding CFF element was a 19-hole tubular ceramic membrane (178 mm length, 3.3 mm channel diameter). The equivalent velocity and specific energy consumption for CFF were also determined using Equations (9) and (10).

As shown in Table 2, the specific energy consumption of RDF is only about 10–30% that of CFF. Although RDF demonstrates clear advantages in energy consumption, several challenges remain for practical wastewater treatment, such as membrane fragility, operating pressure and velocity limitations, equipment complexity, and high investment costs.

### 3.3. Pilot Validation Study for EGC Desulfurization Wastewater

In the pilot validation experiments, both intermittent and continuous operation modes were conducted under optimized conditions (350 rpm, 250 kPa) to evaluate the stability and reliability of the RDF technology.

#### 3.3.1. Intermittent Operation Mode

**Figure 17:**
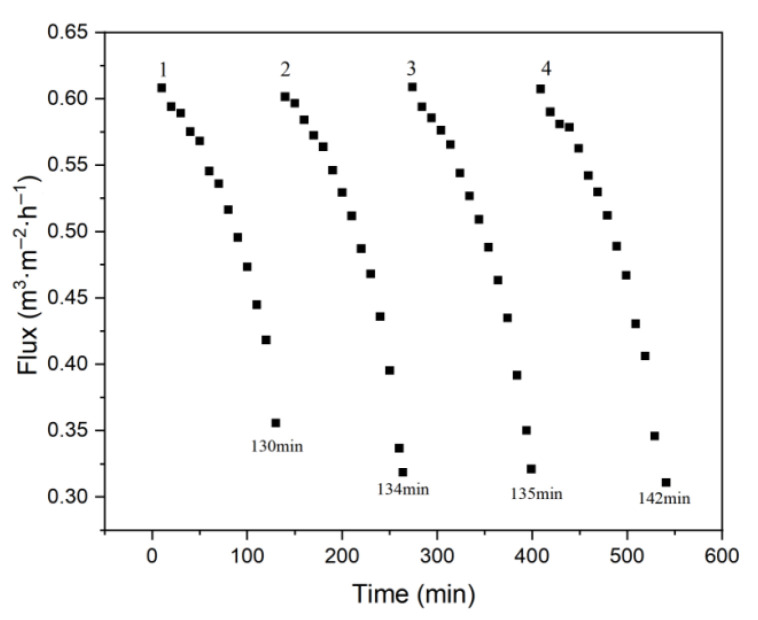
Flux vs. time in the intermittent pilot experiment.

**Figure 18:**
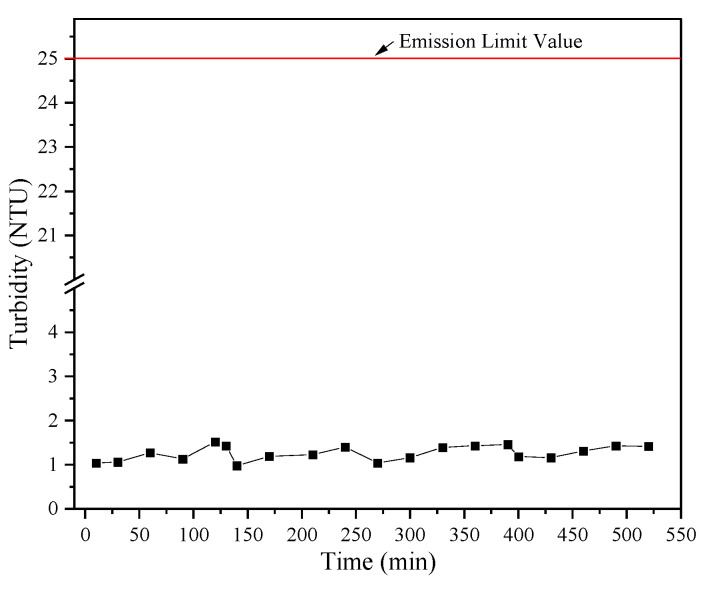
Filtrate turbidity vs. time in the intermittent pilot experiment.

Four intermittent operation experiments were conducted at the pilot site, during which the wastewater in the container was concentrated from 1200 L to 40 L (183 mg·L^−1^ to 5490 mg·L^−1^). The results for flux and turbidity are presented in Figure 17 and Figure 18.

As shown in Figure 17, a 30-fold concentration was achieved in each intermittent operation, with flux gradually declining during the concentration process. The total accumulation time across the four operations was 541 min, and each subsequent run took slightly longer due to increasing membrane fouling.

Figure 18 demonstrates that the permeate turbidity fluctuated but consistently remained below 2.0 NTU, well below the emission limit of 25 NTU. This confirms that the RDF process effectively removed particulates from the wastewater on the pilot scale.

#### 3.3.2. Continuous Operation Mode

**Figure 19:**
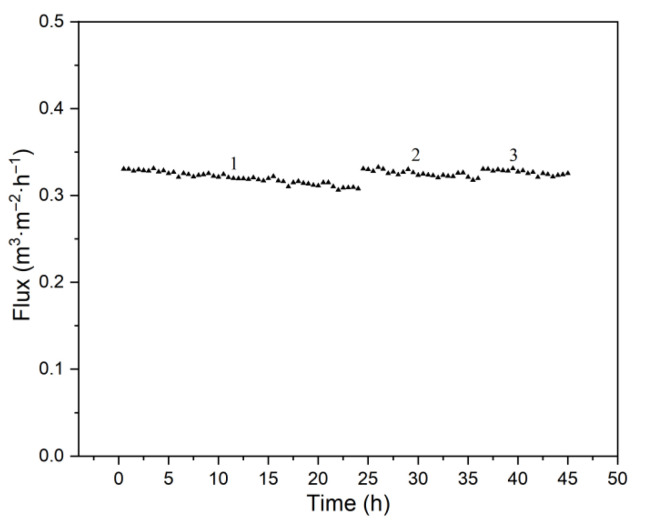
Flux vs. time in the continuous operation pilot experiment.

**Figure 20:**
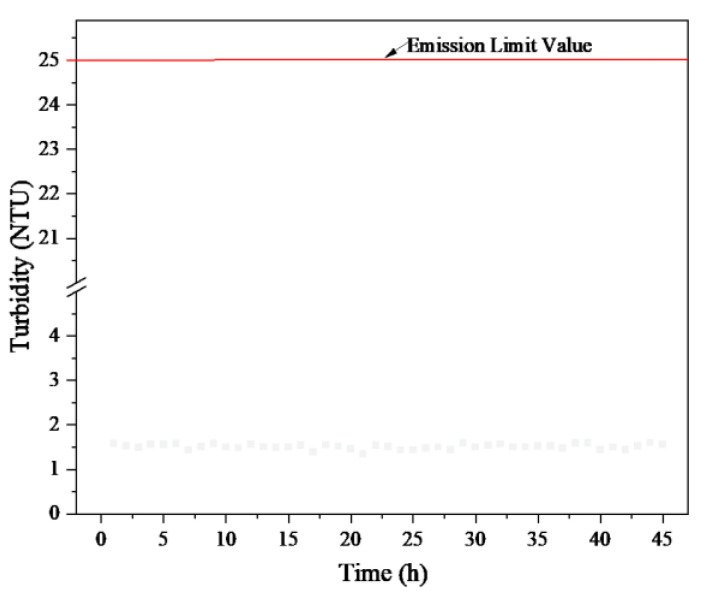
Filtrate turbidity vs. time in the continuous operation pilot test.

Continuous operation is commonly employed to maintain steady operation and a constant treatment capacity in practical applications. The continuous-mode experiment was conducted at 350 rpm and 250 kPa, with the feed, permeate, and concentrated slurry discharge kept constant as the wastewater was concentrated 30-fold. Three long-term continuous runs were performed on-site, with durations of 24, 12, and 9 h, totaling 45 h. The flux and turbidity results are shown in Figure 19 and Figure 20.

During the first run, the initial flux was 0.3305 m^3^·m^−2^·h^−1^, declining to 0.3078 m^3^·m^−2^·h^−1^ after 24 h. Prior to the next filtration, the membrane was soaked and cleaned using the optimized regeneration procedure. The initial flux for the second operation was restored to 0.3308 m^3^·m^−2^·h^−1^, decreasing to 0.3196 m^3^·m^−2^·h^−1^ after 12 h. The third operation started at 0.3287 m^3^·m^−2^·h^−1^ and dropped to 0.3254 m^3^·m^−2^·h^−1^ after 9 h.

Throughout the three continuous operations, flux remained relatively stable. Effective membrane regeneration after cleaning was confirmed. Thus, the RDF process can be implemented continuously for high-concentration-ratio treatment of EGC wastewater.

As shown in Figure 20, filtrate turbidity during continuous operation was slightly higher, likely due to more particulates passing into the permeate at a high concentration. However, all values remained below 2.0 NTU, complying with emission standards.

#### 3.3.3. Comparison of Continuous and Intermittent Modes

In both intermittent and continuous pilot operations, the achieved 30-fold concentration ratio exceeds the 10-fold ratio of tubular CFF. The treated wastewater exhibited a pH of approximately 7.06 and turbidity below 2 NTU (1.26 NTU in intermittent mode and 1.51 NTU in continuous mode), both markedly lower than the 10 NTU turbidity observed for tubular CFF.

The flux in the intermittent mode (0.5333 m^3^·m^−2^·h^−1^) was higher than that in the continuous mode (0.3305 m^3^·m^−2^·h^−1^), and the average turbidity was also lower (1.26 NTU vs. 1.51 NTU, respectively). However, considering operational reliability and automation, the continuous mode under steady-state operation is preferable.

Polycyclic aromatic hydrocarbon (PAH) concentrations in the permeate were measured at 1.19 μg·L^−1^ in the intermittent mode and 1.78 μg·L^−1^ in the continuous mode. The results verify that, in addition to particle removal, the RDF process can also remove PAHs from desulfurization wastewater.

In summary, pilot-scale RDF treatment of EGC wastewater meets the discharge requirements of MEPC.259 (68) established by the IMO.

## 4. Conclusions

This study investigated the separation behavior of EGC desulfurization wastewater using RDF. The permeate flux increased with operating pressure and temperature, decreased with increasing feed concentration, and remained unaffected by the circulation rate. For the 152 mm membrane, flux rose consistently with an increasing rotational velocity. For the 374 mm membrane, flux also increased with rotational velocity at 300 kPa, but at 100 kPa, it increased initially and then declined, reflecting the interplay between enhanced shear force and rising back pressure. Across all experimental conditions, filtrate turbidity met the requirements of MEPC.259 (68).

Unlike CFF, RDF features a velocity and shear force at the membrane surface that both increase with rotational velocity and radius. The enhancement of shear force promotes flux, and this relationship follows a power law. Back pressure induced by centrifugal force within the membrane element is unique to RDF and reduces the effective transmembrane pressure, thus limiting permeate flux. The combined effects of rotation and the membrane size on flux are determined by the balance between shear force and back pressure. To avoid reverse permeate flow, the applied pressure must exceed the maximum back pressure. The specific energy consumption of RDF is only about 10–30% that of CFF.

During pilot-scale validation, RDF achieved a 30-fold concentration of wastewater with filtrate turbidity consistently below 2 NTU, which outperformed CFF (10-fold concentration and 10 NTU turbidity). Continuous operation was found to be preferable for marine applications, providing greater reliability and automation.

Overall, this research presents RDF as an effective and energy-efficient solution for shipboard EGC wastewater treatment, while contributing to a deeper understanding of RDF principles and applications.

## Figures and Tables

**Table 1:** Main parameters of membrane elements used in this study.

Type	Diameter (mm)	Area (m^2^)	Pore Size (μm)	Resistance (10^10^ m^−1^)	Material
1#	152	0.035	0.2	42.94	Al_2_O_3_
2#	374	0.20	0.2	41.93	Al_2_O_3_
3#	152	0.035	0.5	32.70	Al_2_O_3_
4#	374	0.20	0.5	30.99	Al_2_O_3_

**Table 2:** Energy consumption comparison between RDF and CFF.

No.	Rotational Velocity (rpm)	Equivalent Velocity (m·s^−1^)	Specific Energy Consumption (100 kPa)	
			CFF(Wh·m^−3^)	RDF(Wh·m^−3^)
1	300	1.74	3153.12	316.80
2	500	2.91	4733.50	588.00
3	700	4.07	6204.14	1270.38

## Data Availability

Research data is in the manuscript.
